# HTLV-1 is predominantly sexually transmitted in Salvador, the city with the highest HTLV-1 prevalence in Brazil

**DOI:** 10.1371/journal.pone.0171303

**Published:** 2017-02-03

**Authors:** David Nunes, Ney Boa-Sorte, Maria Fernanda Rios Grassi, Graham P. Taylor, Maria Gloria Teixeira, Mauricio L. Barreto, Inês Dourado, Bernardo Galvão-Castro

**Affiliations:** 1 Centro Integrativo e Interdisciplinar de HTLV, Escola Bahiana de Medicina e Saúde Pública, Salvador, Bahia, Brazil; 2 Instituto Gonçalo Moniz, Fundação Oswaldo Cruz/Bahia, Salvador, Bahia, Brazil; 3 Imperial College Healthcare NHS Trust, National Centre for Human Retrovirology, St. Mary’s Hospital, London, United Kingdom; 4 Instituto de Saúde Coletiva, Universidade Federal da Bahia, Salvador, Bahia, Brazil; Centers for Disease Control and Prevention, UNITED STATES

## Abstract

**Background:**

Salvador is the city with the highest number of HTLV-1 infected individuals in Brazil, yet the main route of HTLV-1 transmission is unknown.

**Objective:**

To investigate the association of syphilis infection as a proxy for sexual transmission of HTLV-1 infection in the general population of this city.

**Methods:**

A cross sectional population-based study was conducted with 3,451 serum samples obtained by a representative simple random sampling. Data on gender, age, income, and years of education were collected by questionnaire and the presence of HTLV, HIV and *Treponema pallidum* infection was determined by serology. Logistic regression analysis was used to evaluate the independent effect of the potential explanatory variables to HTLV-1 infection and Odds Ratios (OR) and 95% CI were calculated.

**Results:**

The majority of studied individuals were female (56.4%), had less than 7 years of education (55.3%) and earned two or less minimum wages (52.0%). The overall prevalence of HTLV-1 was 1.48% (51/3,451; 95% CI: 1.10%– 1.94%), which increased with age. Only three persons younger than 17 (3/958; 0.31%; CI 95% 0.06–0.91) years were infected by HTLV-1. Among the 45 syphilis positives, 12 (26.7%) were HTLV positive, while among 21 HIV positives, only one (4.8%) was HTLV positive. HTLV-1 infection was found to be associated with syphilis infection (OR_ADJUSTED_ 36.77; 95% CI 14.96–90.41).

**Conclusion:**

The data presented herein indicate that horizontal transmission between adults is the main route of HTLV-1 infection in the general population of Salvador and that this is likely to occur through sexual contact.

## Introduction

Human T lymphotropic virus type-1 (HTLV-1) is a delta retrovirus of worldwide distribution and it is estimated that at least 5–10 million people harbor the virus [1]. To identify the risk of transmission via tissue transplantation, high prevalence has been defined as greater than 1% in the general population or greater than 1 in 10,000 first time blood donors[2]. The highest areas of prevalence are located in Japan, Africa, the Caribbean Islands, Melanesia, the Mashhad area of northeastern Iran and South America. Brazil, which potentially harbors 800,000 people with HTLV-1, represents the largest number of carriers on the American continent [1, 3].

HTLV-1 is etiologically linked with adult T cell leukemia-lymphoma (ATLL) [4], tropical spastic paraparesis/HTLV-1-associated myelopathy (TSP/HAM) [5, 6], uveitis[7], and infective dermatitis[8]. Furthermore, many other diseases have been associated with HTLV-1 infection, such as polymyositis, sinusitis, broncho-alveolar pneumonia, keratoconjunctivitis sicca and bronchiectasis, indicating multi-systemic involvement [1, 9–11].

The inter-human transmission of HTLV-1 arises from infected lymphocytes, as plasma is unable to promote infection [12] and HTLV-1 viral RNA is either not detected or only detected at very low concentrations in patient plasma[13]. Transmission occurs through contaminated blood or tissue, from mother-to-child, predominantly through breast-feeding, as well as through sexual contact [1, 14]. Since the establishment of blood screening for HTLV-1, the transmission of this virus in endemic areas primarily occurs from mother-to-child and/or through sexual intercourse [15, 16]. The risk of transmission of HTLV-1 per coital act, particularly between new sexual partners, is unknown. However, sexual transmission is more efficient from men to women, with a reported rate of 60.8% compared to 0.4% in the reciprocal direction [17]. In a prospective study of 97 Japanese HTLV-serodiscordant couples followed for five years, the overall transmission rate was approximately 1% per year, and the relative rate of transmission was 3.9 fold higher from infected males to their uninfected female partner than vice versa[18]. The presence of genital ulcers and a higher number of sexual partners increases the risk of sexual transmission of HTLV-1[19].

Salvador, located in the northeastern region of Brazil, is considered an epicenter of HTLV-1 infection in the country, with a prevalence above 1% in the general population [1.74%; (95% CI: 1.1%–2.5%)][20], 1.35% (95%CI: 0.65–2.05%) among blood donors[21] and 0.84% (95% CI; 0.65–1.08) among pregnant women[22]. A previous general population survey found higher rates of HTLV-1 infection among men and women over 50 years compared to those under 50: 6.3% (OR: 12.3; 95% CI: 1.47–103.1) and 9.3% (OR: 9.7; 95% CI: 3.11–30.4) for males and females, respectively. Moreover, no individuals under 13 years of age were found to be infected, suggesting the predominance of sexual transmission of HTLV-1 in this city [20]. In addition, a case control study found that a previous history of sexually transmitted disease (STD) was a risk factor to HTLV-1 infection in blood donors, which is also suggestive of the predominance of sexual transmission [23]. However, blood donors constitute a more biased sample due to the restricted nature of this population, which differs substantially from the general population [24]. In the present study, we aimed to investigate the association of syphilis infection as a proxy for sexual transmission of HTLV-1 infection in the general population of Salvador, a city with an estimated 50,000 HTLV-1 infected individuals.

## Materials and methods

### Study design and population

A cross-sectional study was carried out in order to investigate HTLV-1 infection in the general population of Salvador, located in northeastern Brazil. This city presents a high socio–economic inequality and a population of approximately 2.9 million inhabitants, of which roughly 80% are black or racially mixed African and Portuguese descendants [25]. The dataset was part of a larger biological survey conducted to estimate the incidence and prevalence of the main infectious diseases among residents of all ages of selected neighborhoods called “sentinel areas” in Salvador, from May 1998 to July 2000, as described elsewhere [26]. In short, the study population was drawn from a spatial sample of 30 sentinel areas representing a wide range of living conditions. Each sentinel area, in turn, consisted of two or more adjacent census tracts with similar income levels and basic sanitation coverage [27].

This study analyzed data from 3,451 serum samples, obtained by a simple random sampling procedure without replacement, stored at -20°C. Information on area of residence, sex, age, income, and years of education were collected by a questionnaire. After obtaining signed informed consent, 10 ml of blood were collected from all study participants. For children, blood was collected, after obtaining signed informed consent from their parents or guardian, using appropriate equipment for the child’s age. Samples were anonymized prior to laboratory analysis. HTLV, HIV and *T*. *pallidum* sero-prevalence were determined. The research protocol was approved by both Institutional Research Boards of the Instituto Gonçalo Moniz, Fundação Oswaldo Cruz and of the Escola Bahiana de Medicina e Saúde Pública.

### Laboratory assays

Sera from all volunteers were screened for HTLV 1/2 antibodies by ELISA (HTLV-1 (rp21e enhanced), EIA, Cambridge Biotech Corporation, Worcester, MA, USA); confirmation and discrimination between HTLV-1 and HTLV-2 was performed by western blotting (HTLV Blot 2.4, Genelabs Diagnostics GLD, Science Park Drive, Singapore). HIV antibodies were detected using ELISA (Ortho Diagnostics EIA, Rochester, USA), and confirmed by indirect immunofluorescence (Biomanguinhos, FIOCRUZ, Rio de Janeiro, Brazil), following the manufacturer’s recommendations. *Treponema pallidum* infection was investigated by testing reactivity to non-treponemal cardiolipin using the Venereal Disease Research Laboratory test (Laborclin, Paraná, Brazil), and confirmed by indirect immunofluorescence (FTA, Hoechst/Behring, Germany).

### Data analysis

The outcome variable was prevalence of HTLV-1 infection. Syphilis infection was used as a proxy for sexual transmission of HTLV-1 infection, since other sexual behavior variables were not available in the main survey. Descriptive statistics were determined for each variable. Age was examined in four different strata to analyze trends in HTLV-1 prevalence rates (0–16 yrs; 17–30 yrs; 31–50 yrs and ≥ 51 yrs). Other variables (and cut points) were years of education (>7 years); income (<twice the Brazilian minimum monthly wage (MW), equal to approximately US $125 at the time of survey. Prevalence rates of HTLV-1 infection and respective binomial 95% confidence intervals (CI) pertaining to study variables were estimated. Odds Ratios (OR) and 95% CI from logistic regression analyses were used to evaluate the association of syphilis infection with HTLV-1 infection. The Statistical Package for the Social Science (SPSS, Inc, Chicago, IL) version 14.0 was used for statistical analyses.

## Results

The study population comprised 3,451 subjects of which 1,947 (56.4%) were females; age ranged from 0 to 99 years. Eleven (0.32%) and 23 (0.67%) subjects provided no information about sex and age, respectively. The majority (55.3%; 1895/3425) had less than seven years of education and 52.0% (1660/3192) earned two MW or less.

**Fig 1:**
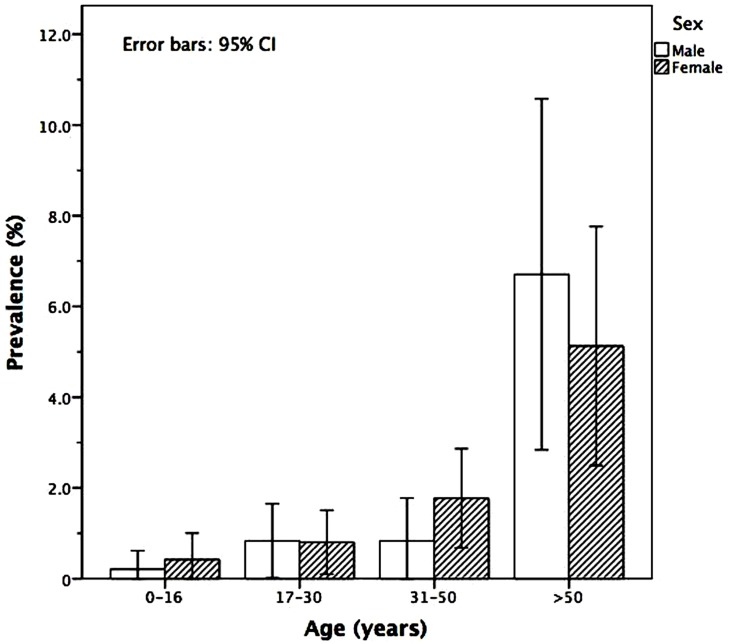
Prevalence of HTLV-1 infection among males and females in Salvador, Bahia-Brazil, stratified by age in years.

The overall prevalence of HTLV-1 was 1.48% (51/3451; 95% CI: 1.10%– 1.94%), which was shown to increase with age (Table 1). HTLV-1 infection rates were 1.27% (19/1,493; 95% CI: 0.77–1.98) among males and 1.59% (31/1,947; 95% CI: 1.08–2.25) in females (Fig 1). Three persons younger than 17 (3/958; 0.31%; CI 95% 0.06–0.91) years were found to be infected by HTLV-1: two girls, aged two and fourteen years, and one 16-year-old boy. Overall prevalence of syphilis and HIV were 1.30% (45/3,451; 95% CI 0.95–1.74) and 0.6% (21/3,451; 95% CI 0.37–0.93), respectively. Among those positive for syphilis, 26.67% (12/45; 95% CI: 14.60–41.94) were positive for HTLV-1, while among the 21 individuals positive for HIV, only one (4.76%; 95% CI: 0.12–23.81) had HTLV-1. Of the 51 HTLV-1 infected individuals, 11 were co-infected with syphilis: 26.32% (5/19) were males, while 19.35% (6/31) were females. One co-infected individual reported no information on sex. The only HIV-infected individual was a 40-year-old female who was HTLV-1 seropositive.

**Table 1:** Study population and Prevalence Rate of HTLV-1 Infection according to study variables. Salvador, Northeast Brazil.

Variable	Study Population		HTLV infection	
	N	%	N (%)	95%CI
**Age**				
0 a 16	958	27.95	03 (0.31)	0.06–0.91
17 a 30	1102	32.15	09 (0.82)	0.37–1.54
31 a 50	928	27.07	13 (1.40)	0.75–2.38
51 +	440	12.84	25 (5.68)	3.71–8.27
Total	3428	100		
**Sex**				
Male	1493	43.4	19(1.27)	0.77–1.98
Female	1947	56.6	31(1.59)	1.08–2.25
Total	3440	100		
**Years of education**				
>7 years	1530	44.67	15 (0.98)	0.55–1.61
≤7 years	1895	55.33	34 (1.79)	1.24–2.50
Total	3425	100		
**Monthly Income**				
>2 MW	1532	47.99	16 (1.04)	0.60–1.69
≤2 MW	1660	52.01	32 (1.93)	1.32–2.71
Total	3192	100		
**Syphilis**				
Negative	3406	98.7	39 (1.15)	0.82–1.56
Positive	45	1.3	12 (26.67)	14.60–41.94
Total	3451	100		
**HIV**				
Negative	3430	99.39	50 (1.46)	1.08–1.92
Positive	21	0.61	01 (4.76)	0.12–23.81
Total	3451	100		

HTLV-1: Human T-lymphotropic virus type 1; CI: Confidence interval; MW: Minimum wage = US$ 125.00 at the time of survey. HIV: Human immunodeficiency virus

HTLV-1 infection was strongly associated with syphilis seroreactivity (OR: 36.77; 95% CI 14.96–90.41) after adjusting for other study variables (Table 2). Males presented an even stronger association (OR: 40.38; 95% CI: 10.30–158.25), which was somewhat less among females (OR 33.41; 95% CI: 8.74–127.68). Additionally, a significant correlation (r = 0.221; p<0.001) was observed between HIV and syphilis (OR _crude_: 44.63; CI95%: 6.17–116.79).

**Table 2:** Crude and adjusted Odds Ratios (OR) regarding the association of HTLV-1 infection and syphilis, adjusted for sex, family income, age, years of education and HIV infection in Salvador, Northeast Brazil.

Variables	OR _crude_ (95% CI)	OR _adjusted_ (95% CI)
Syphilis		
Negative	1.00	1.00
Positive	31.39 (15.09–65.29)	36.77 (14.96–90.41)
Sex		
Male	1.00	1.00
Female	1.26 (0.71–2.23)	1.10 (0.58–2.06)
Age (years)		
0–50	1.00	1.00
51 +	6.65 (3.76–11.74)	9.62 (5.04–18.36)
Family income		
> 2 MW	1.00	1.00
≤ 2 MW	1.86 (1.02–3.41)	2.23 (1.11–4.47)
Years of education		
> 7 years	1.00	1.00
≤ 7 years	1.85 (1.00–3.40)	1.15 (0.57–2.33)
HIV		
Negative	1.00	(*)
Positive	3.38 (0.44–25.67)	

(*) Due to the occurrence of a single case of HIV/HTLV co-infection, it was impossible to perform multivariate modeling

## Discussion

The city of Salvador continues to be an important location for HTLV-1 infection surveillance, as the prevalence among the general population remains above 1% (1.45%). In addition, very few studies involving large samples have investigated HTLV among the general population [24].

Three observations from this study strongly suggest that sexual contact constitutes the main route of HTLV-1 transmission in the general population of Salvador. First, a robust association between syphilis infection and HTLV-1 infection. Those with *T*. *pallidum* seropositivity were almost 40 times more likely to be HTLV-1 positive compared to *T*. *pallidum* soronegative individuals. Second, the seroprevalence of HTLV-1 infection in children and adolescents younger than 17 years was only 0.3%. These HTLV-1 infected persons were two girls, aged two and fourteen years old, and one 16-year-old boy. Third, a sharp increase in the prevalence of HTLV-1 infection in older age groups confirms the adult acquisition of HTLV-1, supporting the previously postulated predominance of sexual transmission [20].

Unprotected sex, multiple partners, history of other sexually transmitted diseases, and sexual intercourse with injection drug users were identified as risk factors for HTLV-1 infection in this city [23, 28]. In addition, the mean age of coitarche, in the state of Bahia at the time of this study, was 15 and 16 years for men and women, respectively [29].

Evidence of sexual transmission as a main route of HTLV-1 has also been reported in the Caribbean [30, 31]. Conversely, breastfeeding has been considered the main route of HTLV-1 transmission in Japan [32]. Although cultural and socioeconomic characteristics are different among Japan and other tropical countries, including Brazil, it is unclear why the major routes of HTLV-1 transmission differ among these countries. We can hypothesize that different methods of contraception could facilitate or prevent the sexual transmission of HTLV-1. The usage of condoms is the preferred method of contraception in Japan, while this is much lower in developing countries, such as Brazil, including in the city of Salvador [23, 33–35]. Accordingly, we have demonstrated that HTLV-1 infection is associated with low family income (OR: 2.23; CI95% 1.11–4.47). Moreover, it is well known that illiteracy and poverty are more frequent in developing areas, and these socioeconomic aspects are also associated with low rates of condom usage [36]. In addition, there is a marked difference between the governmental programs of prevention and health care for people living with HTLV-1 in Japan and Brazil. While these programs are robust in Japan, in Brazil they are incipient, and HTLV-1 infection and its associated diseases are considered neglected diseases [37].

Although sexual transmission was the most probable route of HTLV-1 infection in this study, vertical transmission also plays a role in the dissemination of HTLV-1. Previous studies have shown that the prevalence of HTLV-1 infection among pregnant women in Salvador and other cities in the state of Bahia remains around 1% [22, 38, 39]. Furthermore, it has been claimed that ATLL and IDH are associated with vertical HTLV-1 transmission, while the horizontal route (blood and sexual) is associated with the development of HAM/TSP [8, 40, 41]. In Salvador-Bahia, several cases of both ATLL and IDH have been reported [42–44]

It has been postulated that a period of breastfeeding lasting six months or more is strongly associated with mother-to-child transmission [45]. However, in the early 1980s, the median time of breastfeeding did not exceed more than six months in Brazil [46]. A nationwide Brazilian survey carried out from 1998 to 2008 showed an increase in the median duration of exclusive breastfeeding from 23.4 to 54.1 days and total breastfeeding time of 295.9 to 341.6 days [47]. This has raised great concern regarding the increase of vertical transmission in Brazil, since HTLV antennal screening is not mandatory in this country. Conversely, in Japan, where antennal screening is mandatory, vertical transmission of HTLV-1 is decreasing [48].

In the present study, the prevalence of syphilis in the general population of Salvador was 1.3%, two times higher than the prevalence of HIV-1 (0.6%). Moreover, the prevalence of seropositivity for *T*. *pallidum* among individuals 17–30 years was 1.91%, nine times higher than that of younger individuals ≤ 16 years. Only two individuals aged 15 and 16 years had positive serology, which reinforces the notion that infection by *T*. *pallidum* should be considered a marker of sexual transmission. The higher prevalence in the general population could be explained by the failure of public health programs to control infection. For instance, a recently published editorial estimated that just 12% of sexual partners received treatment for syphilis in Brazil, which is clearly a failure by the public health system to control transmission of syphilis infection [49]. By contrast, Brazil is considered a model among the developing world with respect to the scope and success of its AIDS treatment programs. [50] HIV transmission is relatively controlled in the general population of Salvador, likely as a result of successful government treatment programs, as well as the fact that HIV infection remains restricted to the so-called key populations for this epidemic: mainly men who have sex with men, female sex workers and drug users [51–53].

Preventive measures to avoid HIV and blood-borne infections may have also helped curb HTLV-1 infection in Brazil. However, there is a possibility that new prevention tools based on the treatment of HIV will not be applicable to the prevention of HTLV, potentially leading to higher rates of HTLV-1 transmission.

The present study is limited due to the cross-sectional nature of the data herein; as such, we are unable to determine causal relationships of the observed associations between syphilis infection and HTLV-1. Nevertheless, the observed associations are consistent with previously presented results [20]. In addition, the lack of robust data on major sexual behavior risk factors, such as unprotected sexual intercourse, number of sexual partners and age of coitarche, limits us from definitively asserting that sexual transmission constitutes the main route of HTLV-1 infection in the city of Salvador.

Nonetheless, it is of paramount importance to reinforce preventive measures, such as urging the use of condoms, as well as to implement mandatory HTLV screening for pregnant women throughout Brazil.
